# From the Cochrane Library: Interventions for Mycosis Fungoides

**DOI:** 10.2196/34448

**Published:** 2022-08-26

**Authors:** Alison Kohn Kucharik, Torunn E Sivesind, Jochen Schmitt, Tobias Weberschock, Peggy Wu, Mavra Masood, Robert P Dellavalle

**Affiliations:** 1 Charles E Schmidt College of Medicine Florida Atlantic University Boca Raton, FL United States; 2 Department of Dermatology University of Colorado Anschutz Medical Campus Aurora, CO United States; 3 Center for Evidence-based Healthcare Faculty of Medicine Carl Gustav Technische Universität Dresden Dresden Germany; 4 Department of Dermatology, Venereology and Allergology University Hospital Frankfurt Frankfurt Germany; 5 Working group Evidence-based Medicine Frankfurt Institute for General Practice Goethe University Frankfurt Frankfurt Germany; 6 Department of Dermatology University of California Davis Sacramento, CA United States; 7 Virginia Commonwealth University School of Medicine Richmond, VA United States

**Keywords:** mycosis fungoides, cutaneous T cell lymphoma, CTCL, psoralen plus ultraviolet-A, PUVA, systematic review, lymphoma, cancer, cancer treatment, cancer intervention, Alibert-Bazin syndrome

Mycosis fungoides (MF) is a chronic malignant condition characterized by a proliferation of clonal T helper cells in the skin. MF remains difficult to treat despite being the most common cutaneous T cell lymphoma. The disease is often refractory, with existing treatments providing only a short duration of clinical response [1]. A 2020 Cochrane review, “Interventions for mycosis fungoides,” provides a comprehensive review of evidence from 20 randomized clinical trials of local and systemic interventions for Alibert-Bazin–type MF (N=1369) [2]. Interventions evaluated in this review included topicals, intralesional therapies, phototherapy, total skin electron beam irradiation, radiotherapy, chemotherapy, extracorporeal photochemotherapy (ECP), biologics, and combination therapies.

The authors aimed to assess the efficacy and safety of interventions for MF using two primary outcome measures: health-related quality of life (HRQoL) and adverse events (AEs). Secondary outcomes included complete response (CR) and objective response rate (ORR). A CR was defined as the complete disappearance of all clinical evidence of disease. The ORR was defined as the proportion of patients with a CR or partial response, meaning the regression of measurable disease of at least 50% in the T, N, M, and B categories. Key outcomes are reported in Table 1. HRQoL was only reported in two studies that could not be analyzed together as it was divided by responder versus nonresponder rather than by treatment group. Common AEs ranged from mild symptoms to severe events. Overall, the evidence indicated that the more aggressive therapies (systemic chemotherapy and combination therapies) resulted in more severe AEs. From all therapies, the CR ranged from 0% to 83% (median 31%), and the ORR ranged from 0% to 88% (median 47%).

Data analysis of the five trials assessing the use of psoralen plus UV-A (PUVA) contributed to the key findings of this review, as it is first-line therapy for early-stage MF and is often used as adjunctive treatment in advanced stages. The authors found no evidence to support the addition of bexarotene or intralesional interferon-α (IFN-α) to PUVA when compared to PUVA alone. Separately, they noted that PUVA combined with IFN-α may lead to a higher CR when compared to IFN-α combined with acitretin. The authors did not find evidence to refute the recommendation of PUVA as a first-line treatment. There was insufficient evidence for adjunctive or alternative therapies such as acitretin or ECP to treat MF.

Using GRADE (Grading of Recommendations, Assessment, Development and Evaluations) criteria, the authors report a lack of high-certainty evidence to guide MF treatment. Many trials included in the review were either inadequate in methodological quality, heterogenous in design, or had insufficient sample sizes. Reported outcomes varied across studies, prohibiting conclusive assessments of the safety, efficacy, and HRQoL impact of these interventions.

**Table 1:** Summary of key primary and secondary outcomes.

RCTs^a,b^	Patients, n	Comparison	Anticipated absolute effects (95% CI)^c^	Relative effect (95% CI)	Quality of evidence (GRADE^d^ approach)
1	122	PUVA^e^ vs IFN-α^f^ + PUVA	HRQoL^g^: NM^h^ AEs^i^: NM CR^j^: 731 per 1000 vs 783 per 1000 (636-958) ORR^k^: NM	HRQoL: NM AEs: NM CR: RR^l^ 1.07 (0.87-1.31) (1) ORR: NM	(1) Low
2	16	PUVA vs ECP^m^	HRQoL: NM AEs: Mild nausea after PUVA (n=NR^n^), hypotension in ECP group (n=1) CR: 250 per 1000 vs 50 per 1000 (3-903) ORR: 750 per 1000 vs 53 per 1000 (0-750)	HRQoL: NM AEs: NM CR: RR 0.20 (0.01-3.61) (1) ORR: RR 0.08 (0.01-1.17) (2)	(1) Very low (2) Very low
3	93	PUVA vs PUVA + bexarotene	HRQoL: NM AEs: NM CR: 222 per 1000 vs 313 per 1000 (158-622) ORR: 489 per 1000 vs 460 per 1000 (298-704)	HRQoL: NM AEs: photosensitivity RR 2.68 (0.11-64.04) (1) CR: RR 1.41 (0.71-2.80) (2) ORR: RR 0.94 (0.61-1.44) (3)	(1) Low (2) Low (3) Low
4	82	IFN-α + PUVA vs IFN-α + acitretin	HRQoL: NM AEs: flu-like symptoms 525 per 1000 vs 693 per 1000 (483-987) CR: 700 per 1000 vs 378 per 1000 (245-588) ORR: NM	HRQoL: NM AEs: RR 1.32 (0.92-1.88) (1) CR: RR 0.54 (0.35-0.84) (2) ORR: NM	(1) Low (2) Low
5	27	No maintenance vs PUVA maintenance	HRQoL: NM AEs: NE^o^ CR: NE ORR: NM	HRQoL: NM AEs: NE CR: NE ORR: NM	NR

^a^RCT: randomized controlled trial.

^b^The studies were ordered by summary findings numbers assigned in the original Cochrane review [2].

^c^The risk in the intervention group (and its 95% CI) is based on the assumed risk in the comparison group and the relative effect of the intervention (and its 95% CI).

^d^GRADE: Grading of Recommendations, Assessment, Development and Evaluations.

^e^PUVA: psoralen plus UV-A.

^f^IFN-α: interferon-α.

^g^HRQoL: health-related quality of life.

^h^NM: not measured.

^i^AE: adverse event.

^j^CR: complete response.

^k^ORR: objective response rate.

^l^RR: risk ratio.

^m^ECP: extracorporeal photochemotherapy.

^n^NR: not reported.

^o^NE: not estimable based on reported data.

Although MF, particularly early stage, generally portends a favorable prognosis, a recent cause of death analysis combining all stages of the disease revealed that patients with MF are most likely to die of the disease [3]. The incidence of MF has been increasing over the past 50 years without concurrent improvement in evidence-based treatment options [3]. In line with most MF treatment guidelines, this review supports PUVA as a major intervention used in MF—a therapy that may be limited by a maximum lifetime dose after which increased risk for melanoma and squamous cell carcinoma become a concern [4]. Thus, future efforts should be directed toward high-quality studies with patient-reported outcomes, safety, and efficacy of alternative MF interventions [5].

## Abbreviations

AE: adverse event
CR: complete response
ECP: extracorporeal photochemotherapy
GRADE: Grading of Recommendations, Assessment, Development and Evaluations
HRQoL: health-related quality of life
IFN-α: interferon-α
JAAD: Journal of the American Academy of Dermatology
JID: Journal of Investigative Dermatology
MF: mycosis fungoides
ORR: objective response rate
PUVA: psoralen plus UV-A
